# Control Sequence Ranking for Critical System Based on Health of Equipment Thanks to Choquet Integral

**DOI:** 10.3389/frai.2020.614853

**Published:** 2021-03-24

**Authors:** Mohammed-Farouk Bouaziz, Pascale Marange, Alexandre Voisin, Jean-Francois Petin

**Affiliations:** Universitè de Lorraine, CNRS, CRAN, F-54000 Nancy, France

**Keywords:** decision making, sequences ranking process, health checkup, multiattribute utility theory, choquet intergral

## Abstract

This paper presents a ranking method of operating sequences based on the actual condition of complex systems. This objective is achieved using the health checkup concept and the multiattribute utility theory. Our contribution is the proposal of sequences ranking process using data and experts’ judgments. The ranking results in a decision-making element; it allows experts to have an objective and concise overall ranking to be used for decision making. A case study is presented based on an experimental platform; it allows us to compare two aggregation operators: the weighted mean and the Choquet integral.

## Introduction

In the industrial field, system operating has become increasingly complex. This complexity arises from the following (Zio, 2009):

1 The increasing complexity of controlled systems (dimension, equipment number and heterogeneity, complexity of control, cohabitation of manual and automated equipment, etc.).
2 The difficulty to have a complete view of the system, i.e., mental representation by the plant operators.
3 The increase of imposed constraints (e.g., safety and environmental standards) and induced constraints (e.g., societal).
4 The difficulty for the operators and decision makers to take into consideration all the previous points as a whole.

With the growing consideration to economic, social, and environmental stakes, the safety of critical systems is at the heart of the concerns of specialists, government, and society. As such, it leads to becoming a major issue for both theoretical and applied research. According to the standard (ISO 13849–1:2015 (en)), security can be defined as the ability of an entity to prevent the occurrence, in given conditions, of critical or disastrous events. Therefore, when operating the system, it must be kept in a state in which the risk of human or material damage is limited to an acceptable level. Safety critical systems receive a particular attention since they represent a significant risk at the society level (Devaraj et al., 2020; Kumar et al., 2020; Maurya and Kumar, 2020). Safety critical systems emerge from the interaction of three poles (Tarride, 2013):

1 A complex technical system characterized by several interactions between its subsystems/components with physical couplings and feedbacks.
2 Human operators interacting with the system, in a given environment, with a synthetic vision influenced by several factors (rules, norms, etc.).
3 An environment in which the above two elements evolve and are influenced by.

**FIGURE 1:**
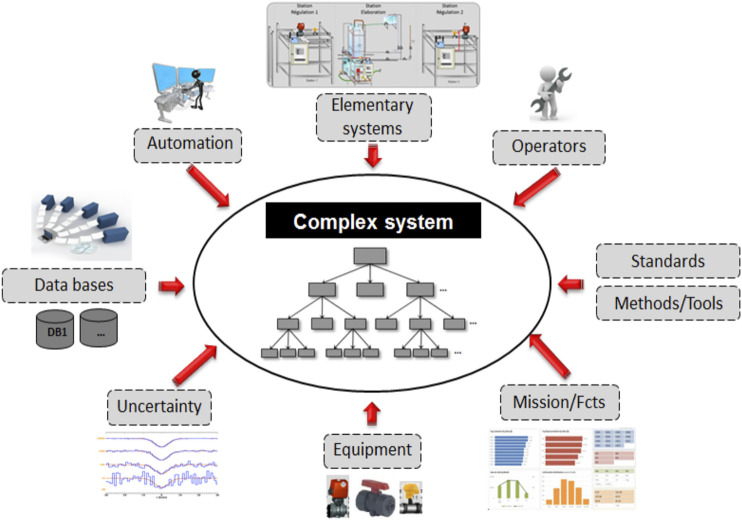
Complex and critical system context.

Despite automation, complex system operating is largely based on human knowledge. This knowledge must consider the interaction of the complex system with its environment as shown in Figure 1. The system is decomposed into subsystems providing a set of functions down to equipment supporting the operation. A successful operating allows the complex system to perform its missions efficiently.

Usually, complex systems require the anticipation of the addressed mission by preparing and defining the operating sequences to be applied, i.e., the sequence of actions to be performed to fulfill the current mission. Such definition has to consider the suitable operating sequences that fulfill the requirement of system security and/or equipment availability. Nevertheless, such task is hardened by the use of huge amount of equipment for other production missions, for safety/maintenance and environment reasons leading to having several possibilities of equipment combination that allow the mission success. Hence, the operating sequence definition is carried out thanks to sequence generating methods at the design stage. Sequence generating methods use a model of the system in order to explore all the possible combinations of equipment state, that will lead to the success of the mission. Once the set of successful sequences is obtained, few of them, considered as the best choice with respect to expert knowledge, are selected and will be used to drive the complex system once in operation. However, in the sequence generation models, the state of the system and its equipment is binary, i.e., ON/OFF. While a sequence can be the best choice when its equipment is fully operating, it may not be the best once equipment is degrading. Furthermore, once in operation, among the set of suitable sequences, the experts must choose the best fitted one based on its knowledge, the actual conditions of the system, etc. Obviously, human experts cannot fully handle the consideration of tens, perhaps few hundreds, of equipment in a sequence neither the gradual performance drift due to equipment degradation. Hence, the motivation of this paper is to propose a ranking of operating sequences from the set of suitable sequences, according to the current state of the system, i.e., health of equipment, with respect to expert’s knowledge.

This paper is organized as follows: sections 2 and 3 are devoted respectively to the study context presentation, the objectives, and the problem statement. Section 4 proposes to introduce the ranking process. Section 5 presents an application of the proposed approach on a case study. Conclusion and perspectives finish this article.

## Study Context

In this article, we consider a set of sequences that should be classified. These sequences are obtained offline or online, using approaches such as verification and validation where on a system model, a property is verified (Schnoebelen et al., 1999; Frey and Litz, 2000; Machado et al., 2006; Lahtinen et al., 2012; Goubali et al., 2016; Gouyon et al., 2020), or synthesis where the model respecting the constraints is obtained by calculation (Wonham and Ramadge, 1987; Ramadge and Wonham, 1989; Yeh and Chang, 2012; Zaytoon and Riera, 2017). How to obtain this set of sequences is not the subject of this paper. Here, we consider that this set contains only the sequence respecting dependability.

In this part, we present the ranking approach to integrate additional information for the sequence selection. While defining a mission, the expert must choose a particular operating sequence to perform from several acceptable sequences. This selection is based on the following:

1 Explicit knowledge: taken into account in the generation of acceptable sequences.
2 Implicit knowledge: for example, the decision criteria defined by the expert.
3 Synthetic knowledge: the representation of complex system by the expert. Indeed, the representation level of expert information is not the same as the sequence actions.
4 Incorrect knowledge: e.g., the plant representation by the expert can be false when the degradation compensation exists.
5 Subjective knowledge: e.g., two experts can select two different sequences.

**FIGURE 2:**
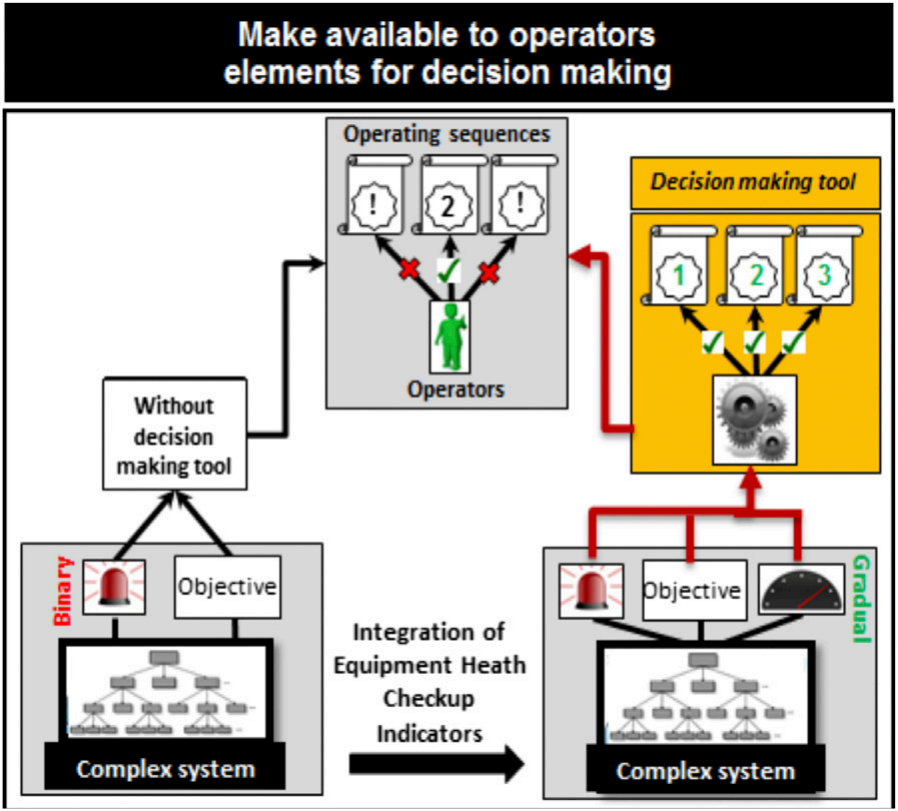
Decision-making context.

Therefore, the approach proposed in this paper aims to help the expert in the selection based on the actual system state. The objective is, firstly, to provide to the expert a ranking of operating sequences based on a set of objective and concise overall information (Figure 2) and, secondly, to make explicit the sequence selection by the following:

1 The definition of the decision criteria.
2 The definition of the decision “rules.”
3 The definition of actual system state.

Our work is original because the approach integrates the following:

1 The equipment characteristics (heterogeneity, number, criticity, etc.).
2 The operational context in the definition of decision criteria (time, cost, performance, solicitation, etc.).
3 The actual equipment conditions (health monitoring).

## Problem Statement

A sequence selection making consists of a ranking of the suitable operating sequences in order to aid the expert. This ranking must take into account the complexity of system operating. This process is based on the system representation through sequences properties and system health state. So, we use the framework of the multicriteria decision making and more specifically the multiattribute utility theory (MAUT). The health checkup provides the current complex state of the equipment of a system in the form of several indicators. These indicators are mapped, thanks to utility functions, on a commensurable scale allowing them to be aggregated. This section presents the concept of system health checkup and introduces the theory of multiattribute utility.

### Health Checkup Concept

Health monitoring aims at following the current state of a system (Kalgren et al., 2006; Omri et al., 2020). The objective of this process is to obtain a representation of the system state and to provide an assessment of its condition/health (Racoceanu, 2006) including incipient degradation. The sensors data are collected to be transformed into indicators, e.g., degradation indicators showing normal or abnormal operating mode of the system according to a reference (Ribot, 2009; Bouaziz et al., 2013).

The concept of equipment health checkup has been addressed in the literature. Byington et al. (2004) indicate that the health checkup corresponds to the actual level of deviations compared to a normal state. Liu (2007) refers by the health of system/equipment its capability to perform the defined and expected function; therefore, health can be considered as the degree of required performance. Shin (2009) indicates that the performance of product/component is measured throughout its period of use, compared to design specifications, by the characterization of this performance degradation over time. Also, Kumar and Pecht (2010) refer to the fact that the context should be integrated within the parameters of health monitoring. Thus, the health vision is a complex concept reflecting functional aspect, dysfunctional aspect, and environmental aspect (Laloix, 2018; Dinh et al., 2020).

**FIGURE 3:**
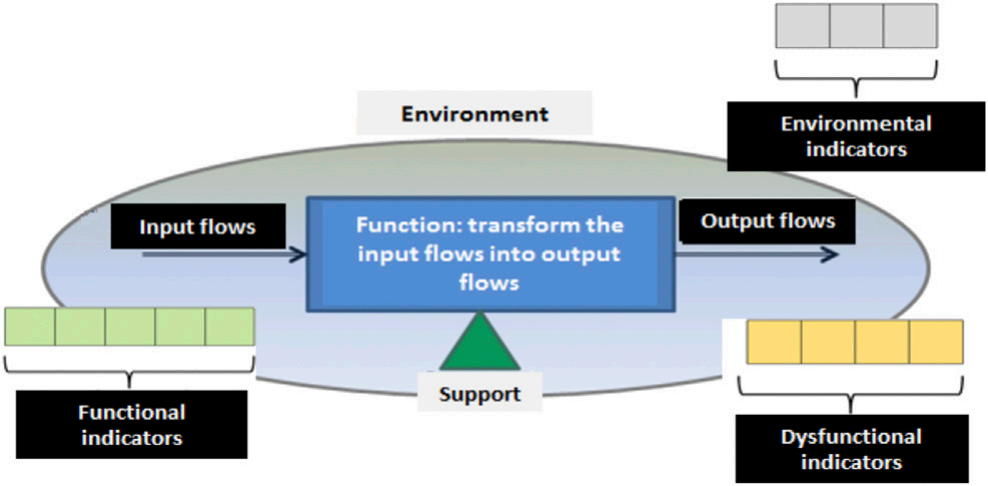
Health checkup associated systemic representation of a system.

Abichou (2013) has proposed a generic representation of these aspects from a systemic representation in the form of health checkup later completed by Laloix (2018) (Figure 3). A health checkup is a set of three classes of indicators:

1 Functional (or performance) indicators: they monitor the function performed by the addressed component at all levels of the hierarchical structure of the system. These indicators are mainly focused on the representation of the function flows (material, energy, and information) and the function performances. We find in this second category in particular i) effectiveness indicators (ratio between results/objectives) and ii) efficiency indicators (ratio between results/resources).
2 Dysfunctional indicators: they are mainly related to the degradation mechanisms evolution. They can represent i) the degradation mechanism (e.g., leak, wear), where indicators are constructed from physical parameters, ii) the observable symptoms (e.g., vibration, temperature increase) and iii) the external degradation factors (e.g., system shocks, mechanical constraints).
3 Environmental indicators: these contextual indicators allow assessing the values taken by functional/dysfunctional indicators in relation to the conditions in which the system evolves. Hence, drift of functional/dysfunctional indicators can be put aside if resulting from operational and environmental conditions change and considered otherwise.

The health checkup formalization of an component *E* is given by Abichou (2013):

$$v\left(E\right)≜\left\{I_{1}^{P},\;\;I_{2}^{P},\ldots ,I_{np_{E}}^{P}\right\}\cup^{\text{​}}\left\{I_{1}^{D},\;\;I_{2}^{D},\ldots ,I_{nd_{E}}^{D}\right\}\cup^{\text{​ }}\left\{I_{1}^{En},\;\;I_{2}^{En},\ldots ,I_{nen_{E}}^{En}\right\}$$ (1)

where v(E) is the set of indicators for a system component *E*, $I_{j}\;$ is the *j*th indicator of performance ($I_{j}^{p}$), degradation ($I_{j}^{D}$), or environment ($I_{j}^{E}$), and np_*E*_, resp. nd_*E*_ and nen_*E*_, stands for the number of performance, resp. degradation and environmental, indicators assigned to E.

**FIGURE 4:**
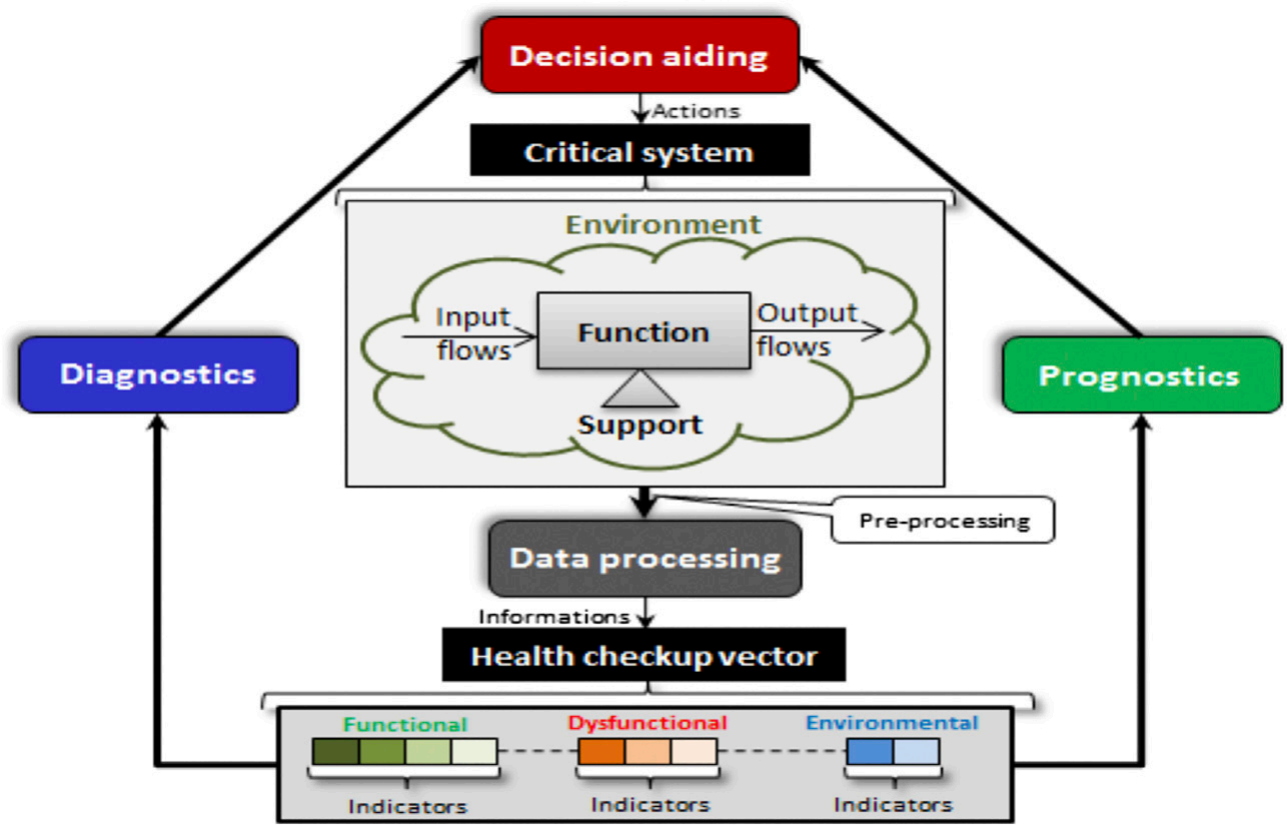
Generic architecture of PHM system.

Prognostics and Health Management approach (PHM) aims at providing support to a system including the monitoring of its real state, the detection of incipient fault, and prediction of impending degradation of a system during its life-cycle (Kalgren et al., 2006). It relies on some important processes such as data processing, diagnostics, prognostics, and decision aiding. Figure 4 presents a typical architecture of PHM and shows the links that exist between these steps and the health checkup. Note that this generic architecture can be adapted to the application needs. The first step involves extracting relevant indicators from a qualitative and quantitative data processing. These indicators can be used for diagnostics to detect, identify, and localize abnormalities; also they can be used for prognostics to estimate the fault evolution. Finally, the decision support step allows choosing an appropriate action plan by evaluating predefined criteria (cost, yield, etc.) (Ben-Daya et al., 2009).

In our work, we are particularly interested in the concept of decision aiding and our study is oriented toward methods and tools from the field of Multicriteria Decision Aiding (MCDA).

### Multiattribute Utility Theory

The problem of sequence ranking is a multicriteria decision problem. For Grabisch and Perny (2007), the performance of each alternative has to be evaluated according to the relevant aspects of the problem; then a classification can be performed following their performances. So, it becomes a comparison problem since the alternatives must be compared 2 to 2 in order to obtain a complete ranking. This comparison is based on the alternative’s representation through criteria values according to the relevant aspects of the problem (Zopounidis and Doumpos, 2002). The difficulty lies in antinomic criteria that vary in opposite way. One classic example is the choice of a car according to its speed and its fuel consumption. Several multicriteria decision-making approaches exist. Among them, the most used are (Öztürk et al., 2005): *PROMETHEE* (*Preference Ranking Organization Method for Enrichment Evaluations*) (Mousavi and Lin, 2020), *ELECTRE* (ELimination Et Choix Traduisant la REalité, i.e,. elimination and choice expressing reality) (Mishra et al., 2020), *AHP* (*Analytic Hierarchy Process*) (Kubler at al., 2016; Liu et al., 2020), *ANP* (*Analytic Network Process*) (Chen et al., 2019), and *MAUT* (Multiattribute Utility Theory) (Dyer, 2005).

Recall that the objective is to classify a set of alternatives (sequences) on the basis of a multicriteria analysis. We use the framework of the MAUT theory, which is one of the most commonly used methods for decision support. We chose MAUT because we believe that taking into account the interaction between criteria is a necessary property in this type of application and a class of operators used in MAUT allows this. In the other methods mentioned above, only ANP and PROMETHEE allow this consideration. However, ANP is very complex to implement and PROMETHEE belongs to a set of methods whose properties are not compatible with our application.

So, according to the MAUT, the problem is formalized as follows. We aim to build a function  f, such as:

$$a≽b=f\left(a_{1},\ldots ,a_{K},b_{1},\ldots ,b_{K}\right)$$ (2)

where a≽b is a binary predicate such as a≽b=1 if a is preferred or indifferent to b. K is the number of decision criteria; $a_{1},\ldots ,a_{K}$ and $b_{1},\ldots ,b_{K}$ represent the decision criteria values for each alternative a and b. ≽ is usually given by an expert as a choice between the two alternatives.

The function f requires the use of aggregation (ψ) and ranking (ϕ) processes. The construction of f can be done by “aggregate and compare” approach or “compare and aggregate” approach (Grabisch and Perny, 2007). The second approach is well fitted when quantitative and qualitative criteria have to be combined or when criteria cannot be mapped to a commensurable scale. Despite these advantages, the “compare and aggregate” approach suffers some limitations (Arrow, 1951; Perny, 1992; Sen, 1986), among which is the loss of transitivity property or the existence of a subset of decisionary criteria. Indeed, Arrow’s theorem (Arrow, 1951) shows that “compare and aggregate” approaches cannot respect, at the same time, the four following conditions for the set of criteria: universality, unanimity, binary independence, and nondictator.

Since our application requires the respect of the four conditions, the former approach has been preferred. The “aggregate and compare” approach also suffers limitations. Among them, Grabisch and Perny (2007) point out 1) the necessity to map the criteria on a common commensurable scale, 2) the conflicts between criteria and possible compensation, and 3) the amount of information requested to set the parameters of the models. Drawbacks 1) and 3) can be overcome since we have expert knowledge as well as operational data in order to provide sufficient information. Limitation 2) remains and cannot be overcome in the “aggregate and compare” approach. It classically happens when using a mean operator. But, using aggregation operator able to capture interaction between criteria allows mitigating this limitation. Nevertheless, such issue is of first importance when dealing with critical systems. Hence, the proposed approach aims at proposing a sequence ranking. The final decision to choose a particular sequence to be operated will remain in the hands of the system operator. The system operator will be able to balance between conflicting criteria and possible compensation. The proposed tool will help him to discard inadequate sequence when considering a huge number of equipment actions to be applied.

The formalization of this approach requires finding a numerical representation of preference, i.e., a function γ :X→ℝ, called score, such as:

∀a and b, a≽b⇔γ(a)≥γ(b) (3)

If ≽ is a complete and transitive binary relation, then γ can be written as:

$$\gamma \left(a\right)=\psi \left(u_{1}\left(a_{1}\right),\ldots ,u_{K}\left(a_{K}\right)\right)$$ (4)

where $u_{i}\;:\;i\to \left(1,K\right)$ are functions of $X_{i}\to \left[0,1\right]$ called marginal utility functions and ψ is an aggregation function (Dimuro at al., 2020). Note that the scale [0 1] is used for utility functions, although in the general case they are defined on ℝ. The $u_{i}^{\prime }\text{s}$ allow the expert to express the acceptable or not acceptable values; utility functions are used to ensure the following:

• A common semantic for decision criteria: the criteria are heterogeneous; for aggregation it is necessary to have the same semantic. It corresponds to a score or satisfaction degree.
• A common scale and commensurability hypothesis: the commensurability ensures that the same utility level, on two different criteria, corresponds to the same satisfaction intensity.

A consequence of formulation of Eq. (4) is that all elements are comparable. This property may not stand. Indeed, some situations may be not comparable according to expert’s preference, i.e., neither a≽b nor b≽a stands. We assume that, for any pair of sequences a and b, the expert is able to compare them and give his preference (a≽b or b≽a). In that sense, $u_{i}$ and ψ reflect the expert preferences.

The aggregation function ψ allows aggregating marginal utilities into a concise overall utility. In order for Eq. 4 to be satisfied, ψ must be idempotent:

ψ (α, α,…,α)=α (5)

with α∈[0 1].

ψ must reflect the preferences of the expert while considering the criteria. Thus, it is desirable that ψ shall reflect the following (Grabisch and Perny, 2007):

1 the relative importance between criteria,
2 the tolerant or intolerant attitudes regarding some criteria,
3 the interactions between criteria

Finally, from Eq. 4, the sequence ranking turns into finding the functions $u_{i}$ and ψ.

### From Health Checkup to Multicriteria Decision

**FIGURE 5:**
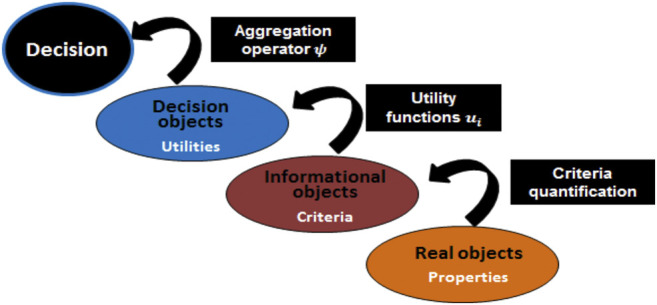
Information transformation in the proposed approach.

**FIGURE 6:**
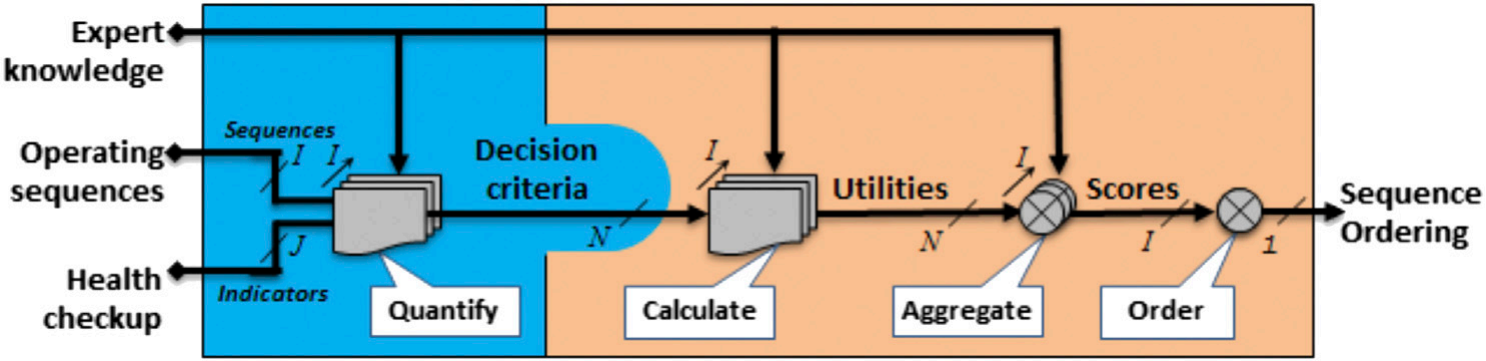
Ranking process.

In this paper, we use the health checkup concept associated with the equipment of operating sequences in combination with MAUT. We propose to order these sequences ($Seq_{i}$) thanks to an aggregation operator that merges some indicators and properties of a sequence into single values that can be ordered. As mentioned in the previous section, the ranking process up to the decision is performed through four steps (see Figure 5). Through these steps, information from health checkup of equipment and sequence properties is transformed toward decision. We present in Figure 5 the information considered along this way. We describe the several information of Figure 6 starting from decision and going to real object properties.

The global score calculation is based on decisional objects described by a set of commensurable and semantically consistent criteria. These criteria are represented by utility functions (see Eq. 4). Hence for a particular sequence, $S\text{e}q_{i}$, Eq. 4 becomes:

$$\gamma \left(S\text{e}q_{i}\right)=\psi \left(u_{1}\left(S\text{e}q_{i}\right),\ldots ,u_{K}\left(S\text{e}q_{i}\right)\right)$$ (6)

To get the K utilities $u_{k\in \left[1\ldots K\right]}$, it is necessary to have an informational representation of the equipment. Hence, this informational representation quantifies decision criteria ($cd_{k}$) on appropriate utility scales:

$$u_{k}\left(S\text{e}q_{i}\right)=u_{k}\left(cd_{k}\left(S\text{e}q_{i}\right)\right)\;\mathrm{for}\;k\in \left[1\ldots K\right]$$ (7)

The criteria are developed from properties of real objects as the properties of a sequence $P\left(Seq_{i}\right),$ or the equipment health checkup indicators $v\left(eq_{j}\right)$:

$$cd_{k}\left(S\text{e}q_{i}\right)=f\left(v\left(eq_{j}\right),P\left(S\text{e}q_{i}\right)\right)$$ (8)

with $eq_{j}\in ℰ\left(Seq_{i}\right)$ and $ℰ\left(Seq_{i}\right)$ the equipment set addressed in $Seq_{i}$.

The last phases of the ranking process, i.e., Eq. 6 and Eq. 7, rely to the classical mathematical framework of MAUT. However, for the first step of quantification, i.e., Eq. 8, the sequence and equipment heterogeneity have to be tackled. In the next part, we present the steps of the ranking process.

## Description of the Ranking Process

The ranking process of operating sequences is divided into four steps (Figure 6):

1 To quantify: a quantification model establishes the link between the sequence properties, health checkup indicators, and decision criteria.
2 To calculate: the decision criteria are mapped into utilities $\;u_{i}$ according to expert’s knowledge.
To aggregate: an aggregation operator ψ computes a global score for each sequence.
3 To order: a ranking operator ϕ to classify all sequences. Before detailing these steps, some assumptions are required:
4 The input operating sequences that belongs to the set of suitable regarding safety aspects.
5 All the input sequences perform the same mission (objective).
6 All the equipment of sequence contributes to the achievement of the mission.
7 The properties and health checkup indicators are available.

### Decision Criteria Quantification

In the general case, quantification maps the operating sequences and equipment condition into decision criteria. The list of criteria is determined from experts’ judgment, in order to identify the main elements taken into account by the expert in his sequence selection (for example, time, cost, and performance indications). For each decision criterion, a mathematical function is calculated to combine the sequence properties and/or the equipment health checkup indicators of a sequence. A key point is to make the quantification step generic enough to be implemented for every operating sequence. Indeed, the sequences may have different number of actions with different components. The output of this step is the values of the N decision criteria.

### Utilities Calculation

A utility function represents the relation between numerical values of the criteria (for example, cost values) and a utility referential from zero (for rejected values) to one (for preferred values). These functions can be obtained by learning from historical data or from experts' knowledge (Grabisch, 2006). As we use expert’s knowledge, the second approach is chosen to define the N utility functions.

### Aggregation

The aggregation mechanism merges information in a global value. In the general case, the aggregate score contributes to decision making from situations that may be contradictory. The aggregation operator ψ (see Eq. 5) associates a score with each sequence from the utility of the criteria.

There are four main classes of aggregation operators (Beliakov et al., 2007): conjunctive, disjunctive, compromise, and hybrids. However, only compromise aggregation operators respect the idempotent property (see Eq. 5). The main compromise operators are arithmetic mean, weighted arithmetic mean, ordered weighted averaging, and fuzzy integral. This last family of aggregation operators takes into account interaction between criteria (Grabisch and Perny, 2007).

Aggregation allows synthesizing the utility values of the criteria in a global score. The choice of an aggregation operator depends on the application (Grabisch et al., 2011). For our application, we decide to compare the calculation results with two compromise operators: weighted arithmetic mean (WA) and the Choquet integral (CI). On one hand, WA operator has been selected since it is a standard operator known by everybody thanks to its widespread use and simplicity. Nevertheless, it cannot be used to model wide spectrum of decision maker preferences since it has intrinsic limitation (Grabish and Labreuche, 2010). On the other hand, CI operator is far less known but has given very good result in several domains and also from a theoretical point of view given raise to several extension (Dimuro at al., 2020). Furthermore, contrary to many aggregation operators, CI is able to handle interactions between criteria and is the only one, in its basic form, to handle homogeneous and heterogeneous interrelationships (Sun et al., 2018). Such interaction should be understood not as correlation between entries but as modeling the dependencies between the criteria thanks to the preference of the decision maker (Marichal, 2000a).

The WA operator associates a different weight with each criterion, and the sum of these weights is equal to 1. The formulation of the WA is given by:

$$WA\left(x_{1},\ldots ,x_{n}\right)=\overset{n}{\underset{i=1}{\sum }}W_{i}.x_{i}\;with:\;\overset{n}{\underset{i=1}{\sum }}W_{i}=1$$ (9)

The CI operator uses parameters which reflect the criteria weights and the interaction degree between these criteria. These parameters are represented in the form of capacity (or fuzzy measures) (Marichal, 2000b). The CI is defined by Murofushi and Sugeno (1991) as:

$$CI\left(x_{1},\ldots ,x_{n}\right)=\overset{n}{\underset{i=1}{\sum }}\left(x_{\left(i\right)}-x_{\left(i-1\right)}\right).\mu \left(A_{i}\right)\;$$ (10)

where $x_{\left(1\right)},\ldots ,x_{\left(n\right)}$ represents the normalized values of the criterion, where (.) is a permutation operator such that $x_{\left(1\right)}\leq x_{\left(2\right)}\leq \ldots \leq x_{\left(n\right)}$, with $x_{\left(0\right)}=0$, and $A_{i}=\left\{i,\ldots ,n\right\}$. $X\;:\left\{X_{1},\ldots ,X_{n}\right\}$ are the normalized values. μ is the capacity. μ(A) represents the importance degree of the set A⊆X in the computation of the global value X.

An analysis of the capacity can be carried out using the Shapley importance index and the interaction index (Marichal, 2000a). Shapley index takes into account the mean importance of a criterion in relation with its contributions for all capacity. In a similar manner, the interaction index quantifies the interaction between two criteria on all capacity.

### Ranking

The last step of the ranking process aims at classifying the scores γ from the best to the lowest. Since the aggregation computes the scores γ on ℝ, the comparison operator ≥  is used to classify them (see Eq. 3). This aspect must reflect the ranking represented by the preference relation ≽. The result is an element of decision making for the definition of the operating sequence.

## Case Study: Application to CISPI^a^ Critical Subsystem

**FIGURE 7:**
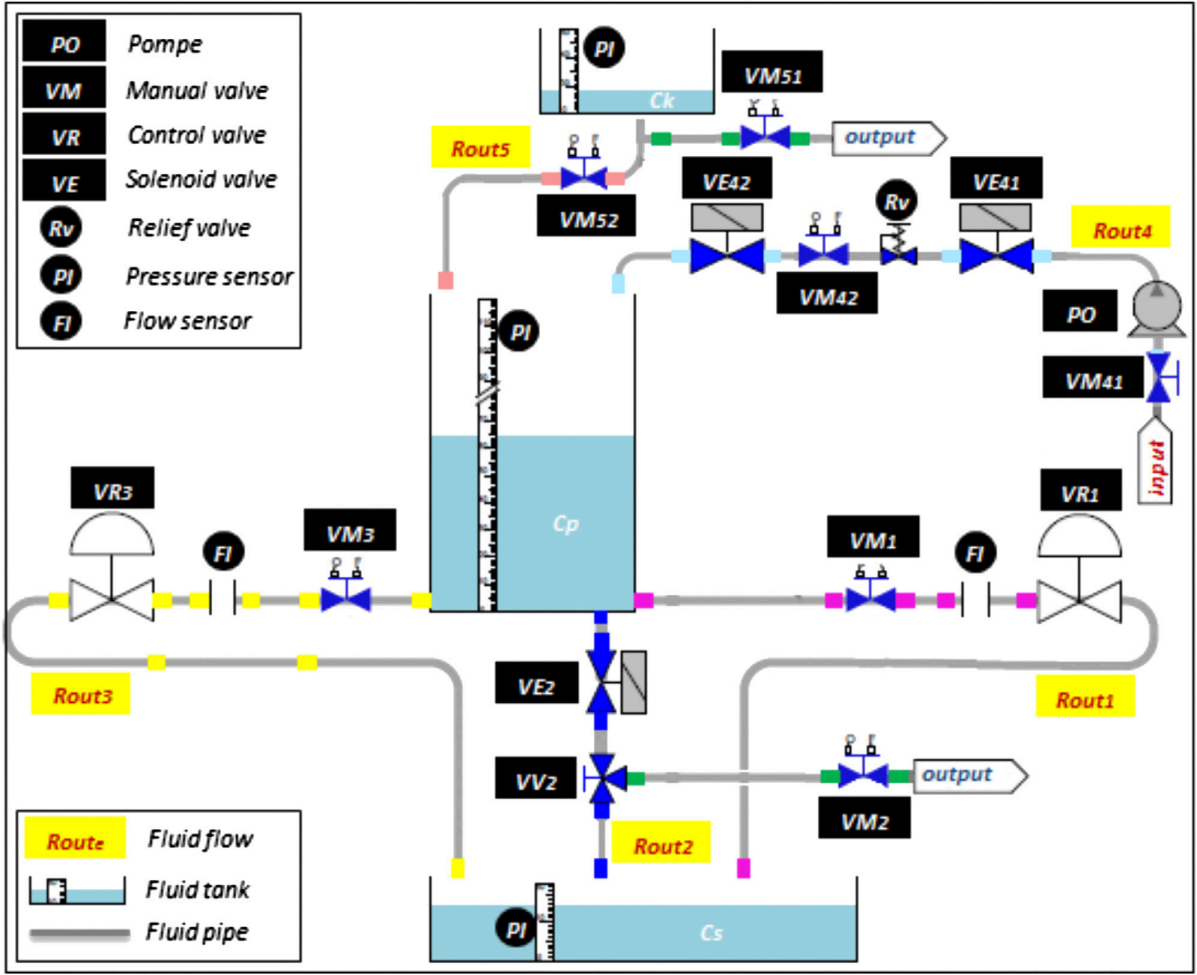
CISPI platform.

Among Safety Critical Systems (SCS) (Diaz et al., 2020; Wang et al., 2017), we are interested in those related to the chemical or nuclear industry. In these industries, the problem of piloting amounts to choosing the best lineage according to several criteria of different natures. CISPI, a research platform of the CRAN (Centre de recherche en automatique de Nancy) scales down a subsystem of a power genetor plant. Figure 7 shows the operating subsystem which controls the liquid flow through the *C*_*k*_ *, C*_*p*_, and *C*_*s*_ tanks. Note that the objective mission is to fill the storage tank *C*_*s*_. This circuit is composed of 1) three routes: *R*_*1*_ *, R*_*2*_,and *R*_*3*_ between the *C*_*p*_ and the *C*_*s*_ tanks, 2) two linear valves *VR*_*1*_ and *VR*_*3*_ to control the flow of *R*_*1*_ and *R*_*3*_, 3) solenoid valve *VE*_*2*_ to control the *R*_*2*_ flow, and 4) four manual valves *VM*_*1*_ *, VM*_*2*_ *, VV*_*2*_, and *VM*_*3*_.

To ensure a sufficient level of liquid in the *C*_*p*_ tank, two additional routes are solicited *R*_*4*_ and *R*_*5*_. From an external *input* source, *R*_*4*_ allows filling the tank *C*_*p*_ through a pump *PO*, two solenoid valves *VE*_*41*_, *VE*_*42*_, and two manual valves *VM*_*41*_ and *VM*_*42*_. A relief valve *Rv* limits the internal pressure of *R*_*4*_ in order to protect equipment. *R*_*5*_ ensures the same mission as *R*_*4*_; it is associated with a storage tank *C*_*k*_ and controlled by two manual valves *VM*_*51*_ and *VM*_*52*_. Finally, CISPI is instrumented through pressure sensors *PI* and flow sensors *FI*.

For the CISPI installation (Figure 7), the elementary lineages are as follows (Clanché et al., 2010):

1 Lineage N°1: filling up with Cp tank and input source (R4 and R1/2/3)
2 Lineage N°2: filling up with Cp and C_*k*_ tanks (R5 and R1/2/3)
3 Lineage N°3: filling up with *C*_*p*_, *C*_*k*_ tanks and *input* source (*R*_*4/5*_ and *R*_*1/2/3*_)

When performing a sequence, the system operator must handle several equipment and move between manual equipment with estimated execution times *ET*_*p*_ (Table 1).

**TABLE 1:** Execution time (in time unit *TU*).

	*VV2*	*VM1*	*VM2*	*Dock*
*VV2*		15	15	10
*VM1*	15		30	10
*VM2*	15	30		10

For the two routes of the subsystem, three sequences are defined for the target mission as follows:

1 $Seq_{1}$: ┴VM1 ┴VR1 (R1)
2 $Seq_{2}$: ┬VM2 ┴VV2 ┴VE2 (R2)
3 $Seq_{3}$: ┴VM1 ┴VR1 ┬VM2 ┴VV2 ┴VE2 (R1 and R2)

where ┴ and ┬ stand for the valve opening and closing actions.

**FIGURE 8:**
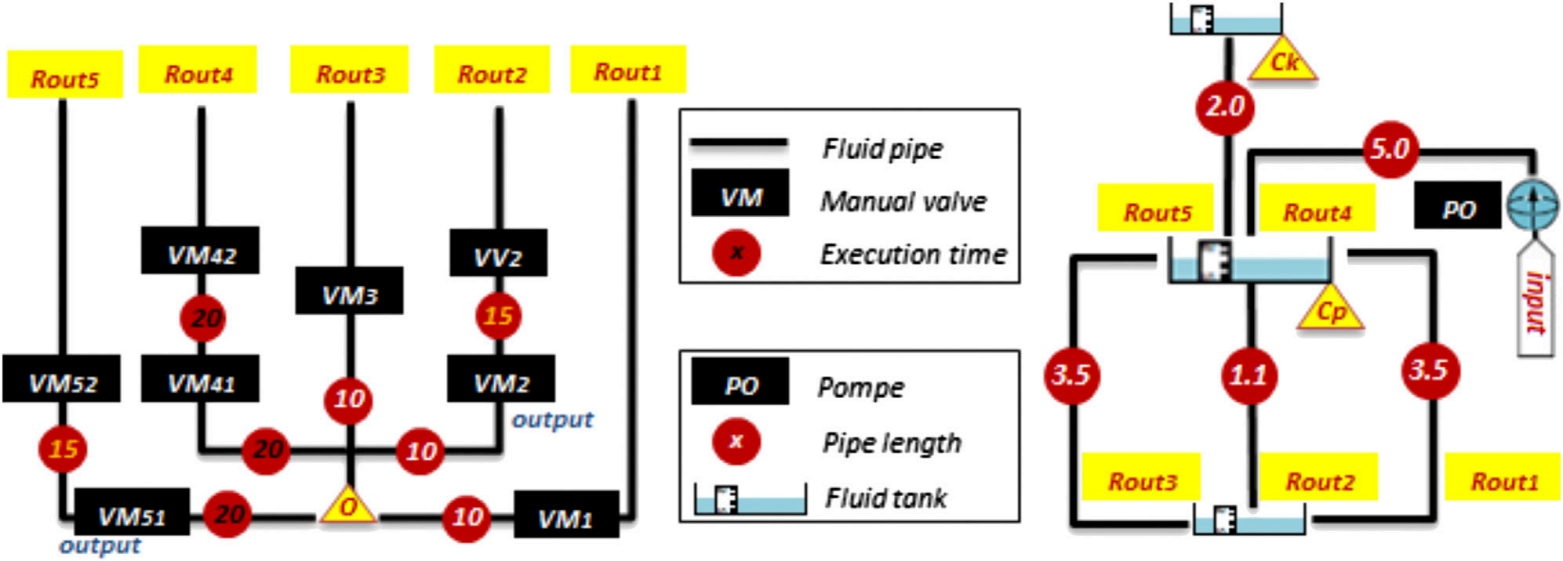
Execution time and pipe length.

First of all, the criteria of Table 2 are considered. Note that each decision criterion combines the properties of the operating sequence and the health checkup of equipment. For example, Figure 8 shows respectively the execution time (in time unit UdT) and the pipe length (in length unit LU).

**TABLE 2:** Decision Criteria.

	*Definition*	*Qualitative link*	*Quantitative link*
cd_1_(Seq_i_)	The duration of the completion of the sequence	f (*V*(eq_j_),*P*(Seq*_i_*))	cd_1_(Seq_i_) = *f* (ET_o_, ET_f_,ET_p_)
cd_2_(Seq_i_)	The operating sequence costs	f (*V*(eq_j_),*P*(Seq_*i*_))	cd_2_(Seq_i_) = *f* (CAM, CdM)
cd_3_(Seq_i_)	The percentage of automatic actions	f (*P*(Seq_*i*_))	cd_3_(seq_i_) = *f* (%AA)
cd_4_(Seq_i_)	The performance of the task achievement	f (*V*(eq_j_),*P*(Seq_*i*_))	cd_4_(Seq_i_) = *f* (Volume,D_n_,D_f_)

Each sequence is characterized by a set of properties associated with the actions (manual/automatic) and the direct equipment (as valve, pump) or the structure equipment (as tanks and pipes).

In the proposed model, the equipment actual condition is taken into account, by health checkup $\;v\left(eq_{i}\right)$ concept. As defined above, the equipment health checkup includes three classes of indicators: functional, dysfunctional, and environmental. The $\;v\left(eq_{i}\right)$ indicators for VM_i_, T_x_, and C_x_ are summarized in Table 3. These indicators are defined by the available measurements and/or expert’s knowledge.

**TABLE 3:** Health checkup indicators.

		Elements	Value/Unit
*VM*_*1*_	Perf	Opening/closing time $T_{o},T_{f}$	5*UdT*
		Manual actions cost CAM	7*Udc*
		Materials cost CdM	5*Udc*
	Dysf	Blocking opening	-
	Envir	Height of liquid *L*_*i*_	*M*
*T*_*x*_	Perf	Nominal flow *D*_*n*_	*m3/s*
	Dysf	Leak flow *D*_*f*_	*m3/s*
	Envir	External *T°*	*°c*
*C*_*X*_	Perf	Nominal flow *D*_*n*_	3.5 10–3 *m* ^*3*^ */s*
	Dysf	Leak flow *D*_*f*_	*m3/s*
	Envir	Internal *T°*	*°c*

For our academic example, we consider the following numerical value:

1 Tank surface: S_CS_ = 0.16 m^2^; S_CP_ = 0.49 m^2^
2 Height of the tank Cp: L_CP_ = 0.80 m
3 Heights difference in the tank Cs: L_CS_ = 0.20 m; volume to fill V_MO_ = 0.2 * 0.49 = 0.098 m^3^
4 Length, diameter, and surface of the pipes: T_L1_ = 3.55 m; T_L2_ = 1.15 m; D_T_ = 40*10–3 m; S_T_ = 12.56*10–4 m^2^
5 Average speed: Sa_CP_ = 2*g*L_CP_/2 = 7.848 m/s; nominal flow: Dn_T_ = Sa_CP_* D_T_ = 9.89 *10–3 m^3^/s
6 Flight flow: D_n_ = 9.89 10–4 m^3^/s
7 Costs of manual actions and operating cost equipment (CAM; CdM) = (7; 5) UdC (cost unity)
Costs of automatic actions and operating cost equipment (CAA; CdM) = (1; 3) UdC
8 Opening/closing time of manual valve (2positions): ET_o_ = ET_f_ = 5UdT (Time unity)
9 Opening/closing time of manual valve (3positions): ET_o_ = ET_f_ = 7UdT
10 Opening/closing time of automatic valve: ET_o_ = ET_f_ = 3UdT

Note that the initial state corresponds to the plan shutdown; this allows us to assume the initial state of the operated equipment when defining the sequences. For this critical system, we assume also that even if equipment was initially closed, closing action must be verified. From the three lineages presented above, 21 operating sequences are defined. These sequences are assumed qualified from safety point of view and optimal (i.e., no useless action). The set of acceptable sequences are described in Table 4. We use “$Seq_{i}$” to refer to a specific sequence.

**TABLE 4:** Complete set of operation sequences.

			*Route #*	*Operating sequence*	*Number of action*
CISPI	*Lineage N°1*	*Seq_1_*	{*R4,R1*}	┴{*VM_41_*,*VM_42_*} ┴{*VE_41_,VE_42_*} ┴{*PO*} ┴{*VM_1_*} ┴{*VR_1_*}	*┬* : *0*
					*┴* : *7*
		*Seq_2_*	{*R4,R2*}	*┴*{*VM_41_*,*VM_42_*} *┴*{*VE_41_,VE_42_*} *┴*{*PO*} *┬*{*VM_2_*,*VV_2_*} *┴*{*VE_2_*}	*┬* : *1*
					*┴* : *7*
		*Seq_3_*	{*R4,R1,R2*}	*┴*{*VM_41_*,*VM_42_*} *┴*{*VE_41_,VE_42_*} *┴*{*PO*} *┴*{*VM_1_*} *┴*{*VR_1_*} *┬*{*VM_2_*} *┴*{*VV_2_*} *┴*{*VE_2_*}	*┬* : *1*
					*┴* : *9*
		*Seq_4_*	{*R4,R3*}	*┴*{*VM_41_*,*VM_42_*} *┴*{*VE_41_,VE_42_*} *┴*{*PO*} *┴*{*VM_3_*} *┴*{*VR_3_*}	*┬* : *0*
					*┴* : *7*
		*Seq_5_*	{*R4,R1,R3*}	*┴*{*VM_41_*,*VM_42_*} *┴*{*VE_41_,VE_42_*} *┴*{*PO*} *┴*{*VM_1_,VM_3_*} *┴*{*VR_1_,VR_3_*}	*┴* : *0*
					*┴* : *9*
		*Seq_6_*	{*R4,R1,R3*}	*┴*{*VM_41_*,*VM_42_*} *┴*{*VE_41_,VE_42_*} *┴*{*PO*} *┬*{*VM_2_*} *┴*{*VV_2_,VM_3_*} *┴*{*VE_2_,VR_3_*}	*┴* : *1*
					*┴* : *9*
		*Seq_7_*	{*R4,R1,R2,R3*}	*┴*{*VM_41_*,*VM_42_*} *┴*{*VE_41_,VE_42_*} *┴*{*PO*} *┬*{*VM_2_*} *┴*{*VM_1_*,*VV_2_,VM_3_*}	*┬* : *1*
					*┴* : *11*
	*Lineage N°2*	*Seq_8_*	{*R5,R1*}	*┬*{*VM_51_*} *┴*{*VM_52_*,*VM_1_*} *┴*{*VR_1_*}	*┬* : *1*
					*┴* : *3*
		*Seq_9_*	{*R5,R2*}	*┬*{*VM_2_,VM_51_*} *┴*{*VM_52_,VV_2_*} *┴*{*VE_2_*}	*┬* : *2*
					*┴* : *3*
		*Seq_10_*	{*R5,R1,R2*}	*┬*{*VM_51_,VM_2_*} *┴*{*VM_52_,VM_1_,VV_2_*} *┴*{*VR_1_*,*VE_2_*}	*┬* : *2*
					*┴* : *5*
		*Seq_11_*	{*R5,R3*}	*┬*{*VM_51_*} *┴*{*VM_52_*,*VM_3_*} *┴*{*VR_3_*}	*┬* : *1*
					*┴* : *3*
		*Seq_12_*	{*R5,R1,R3*}	*┬*{*VM_51_*} *┴*{*VM_52_*,*VM_1_,VM_3_*} *┴*{*VR_1_,VR_1_*}	*┬* : *1*
					*┴* : *5*
		*Seq_13_*	{*R5,R2,R3*}	*┬*{*VM_51_,VM_2_*} *┴*{*VM_52_*,*VV_2_,VM_3_*} *┴*{*VE_2_,VR_3_*}	*┬* : *2*
					*┴* : *5*
		*Seq_14_*	{*R5,R1,R2,R3*}	*┬*{*VM_51_,VM_2_*} *┴*{*VM52*,*VM_1_*,*VV_2_,VM_3_*} *┴*{*VR_1_,VE_2_,VR_3_*}	*┬* : *2*
					*┴* : *7*
	*Lineage N°3*	*Seq_15_*	{*R4,R5,R1*}	*┬*{*VM_51_*} *┴*{*VM_41_*,*VM_42_*} *┴*{*VE_41_,VE_42_*} *┴*{*PO ┴*{*VM_52_*,*VM_1_*} *┴*{*VR_1_*}	*┬* : *1*
					*┴* : *8*
		*Seq_16_*	{*R4,R5,R2*}	*┬*{*VM_2_,VM_51_*} *┴*{*VM_41_*,*VM_42_*} *┴*{*VE_41_,VE_42_*} *┴*{*PO*} *┴*{*VM_52_,VV_2_*} *┴*{*VE_2_*}	*┬* : *2*
					*┴* : *8*
		*Seq_17_*	{*R4,R5,R1,R2*}	*┬*{*VM_51_,VM_2_*} *┴*{*VM_41_*,*VM_42_*} *┴*{*VE_41_,VE_42_*} *┴*{*PO*} *┴*{*VM_52_,VM_1_,VV_2_*} *┴*{*VR_1_*,*VE_2_*}	*┬* : *2*
					*┴* : *10*
		*Seq_18_*	{*R4,R5,R3*}	*┬*{*VM_51_*} *┴*{*VM_41_*,*VM_42_*} *┴*{*VE_41_,VE_42_*} *┴*{*PO*} *┴*{*VM_52_*,*VM_3_*} *┴*{*VR_3_*}	*┬* : *1*
					*┴* : *8*
		*Seq_19_*	{*R4,R5,R1,R3*}	*┬*{*VM_51_*} *┴*{*VM_41_*,*VM_42_*} *┴*{*VE_41_,VE_42_*} *┴*{*PO*} *┴*{*VM_52_*,*VM_1_,VM_3_*} *┴*{*VR_1_,VR_3_*}	*┬* : *1*
					*┴* : *10*
		*Seq_20_*	{*R4,R5,R2,R3*}	*┬*{*VM_51_,VM_2_*} *┴*{*VM_41_*,*VM_42_*} *┴*{*VE_41_,VE_42_*} *┴*{*PO*} *┴*{*VM_52_*,*VV_2_,VM_3_*} *┴*{*VE_2_,VR_3_*}	*┬* : *2*
					*┴* : *10*
		*Seq_21_*	{*R4,R5,R1,R2,R3*}	*┬*{*VM_51_,VM_2_*} *┴*{*VM_52_*,*VM_41_*,*VM_42_*} *┴*{*VE_41_,VE_42_*} *┴*{*PO*} *┴*{*VM_1_*,*VV_2_,VM_3_*} *┴*{*VR_1_,VE_2_,VR_3_*}	*┬* : *2*
					*┴* : *12*

┴: opening action / ┬: closing action

Each sequence refers to a route leading to a set of operated equipment, sensors, and structure equipment. This equipment is assessed by the health checkup indicators. Thus, the sequence properties and health check up indicators are the parameters to choose a sequence to be performed.

### Decision Criteria Quantification

For this study, criteria were defined for the ranking. Each decision criterion *cd* takes as argument either the sequence properties or the sequence properties and the health check up indicators of the equipment as follows (Table 4).

The time of the sequence achievement is calculated according to the opening/closing time $(ET_{o}$ and $ET_{f}$) of the equipment $\left(eq_{j}\right)$ for actions $\left(A_{i}\right)$, and execution time $\left(ET_{P}\right)\;$ between manual equipment $\left(eq_{j}^{M}\right)$ starting from an initial position $I_{0}$. $cd_{1}$ is given as follows:

$$cd_{1}\left(S\text{e}q_{i}\right)=\underset{A_{j}\in S\text{e}q_{i}}{\sum }ET\left(A_{j}\right)+\overset{{\left|{\text{Seq}}_{\text{i}}\right|}^{\text{man}}\;}{\underset{j=0}{\sum }}ET_{p}\left(eq_{j}^{M},eq_{j+1}^{M}\right)\;$$ (11)

with ${\left|Seq_{i}\right|}^{man}$ the number of manual actions. $ET\left(A_{j}\right)\in \left\{ET_{o}\left(eq_{j}\right),\;ET_{f}\left(eq_{j}\right)\right\}$ and $\left\{eq_{0}=eq_{{\left|Seq_{i}\right|}^{man}+1}=I_{0}\right\}$.

The second criterion takes into account the costs of manual and automatic actions ($CAM_{j},CAA_{j}$) and material costs CdM for given equipment $eq_{j}$:

$$cd_{2}\left(S\text{e}q_{i}\right)=\overset{\left|{\text{Seq}}_{\text{i}}\right|}{\underset{j=1}{\sum }}\left(CA_{j}+CdM\left(eq_{j}\right)\right)$$ (12)

with $\left|Seq_{i}\right|$ the number of actions in the sequence and $CA_{j}\in \left\{CAM_{j},CAA_{j}\right\}$.

The third criterion is the percentage of automated actions in the sequence. This criterion depends on the number of automatic actions:

$$cd_{3}\left(S\text{e}q_{i}\right)=\frac{{\left|{\text{Seq}}_{\text{i}}\right|}^{auto}\;}{\left|{\text{Seq}}_{i}\right|}$$ (13)

with ${\left|Seq_{i}\right|}^{auto}$ being the cardinality of automatic actions.

Finally, the fourth criterion is the performance of the task achievement. This criterion is based on the tanks volumes V_MO_, lineage volumes V_Lg_, nominal flows D_n_, and leak flows D_f_ for all equipment. We assume that the initial levels are sufficient to achieve the mission:

$$cd_{4}\left(S\text{e}q_{i}\right)=\frac{V_{MO}\left(eq_{j}\right)\;}{\sum_{l=1}^{\left|Ln\right|}D_{n}\left(Eq_{j}\right)+\sum_{j=0}^{\left|{\text{Seq}}_{\text{i}}\right|}D_{f}\left(eq_{j}\right)}+\overset{\left|{\text{Seq}}_{\text{i}}\right|}{\underset{j=0}{\sum }}\frac{V_{Lg}\left(eq_{j},eq_{j+1}\right)}{D_{n}\left(eq_{j}\right)+\sum_{j=0}^{\left|{\text{Seq}}_{\text{i}}\right|}D_{f}\left(eq_{j}\right)}\approx \frac{V_{MO}\left(eq_{j}\right)}{\sum_{l=1}^{\left|Ln\right|}D_{n}\left(Eq_{j}\right)+\sum_{j=0}^{\left|{\text{Seq}}_{\text{i}}\right|}D_{f}\left(eq_{j}\right)}\;$$ (14)

with $\left(L_{d}-L_{s}\right).\frac{Scs}{L_{p}.Scp}\leq 1$, ($S_{i}$, $L_{i}$) being the surfaces and the levels of $C_{p}\;$ and $C_{s}$ tanks. $L_{d}\;$ is the desired level. In addition, the volume V_MO_ is much higher than the volumes in the various equipment $\overset{\left|Seq_{i}\right|}{\underset{j=0}{\sum }}V_{Lg}\left(eq_{j},eq_{j+1}\right)$.

For each sequence, the value of each criterion must be calculated from the numerical values given. Obtaining the values of each criterion for the first sequence will be detailed, and the criteria values of the other two sequences will be given directly.

If we replace the numerical values of these parameters, we obtain the following results for the first sequence Seq_1_:

$$Seq_{1}:\{\begin{array}{c} \begin{array}{c} CD_{1}=T\left(VM1\right)+T\left(VR1\right)+T_{p}\left(dock,\;VM1\right)+T_{p}\left(VM1,\;dock\right)\\ CD_{2}=CA\left(VM1\right)+CA\left(VR1\right)+CdM\left(VM1\right)+CdM\left(VR1\right) \end{array}\\ \begin{array}{c} CD_{3}=\frac{{\left|{\text{Seq}}_{1}\right|}^{auto}\;}{\left|{\text{Seq}}_{1}\right|}\\ CD_{4}=\frac{{\text{V}}_{\text{MO}}}{Dn}, \end{array} \end{array}$$

$$Seq_{1}:\{\begin{array}{c} \begin{array}{c} cd_{1}=\left(5+3)+(10+10\right)=28UdT\\ cd_{2}=\left(7+1)\;+(5+3\right)=16UdC \end{array}\\ \begin{array}{c} cd_{3}=1/2=0.5\\ cd_{4}=\frac{0.098}{9.86*10^{-3}}=9.94s \end{array} \end{array}.$$

Now we replace the numerical values of these parameters. The criteria are calculated for some sequences and are shown in Table 5.

**TABLE 5:** Learning subset of sequences and score given by the expert.

	*cd*_*1*_	*cd*_*2*_	*cd*_*3*_	*cd*_*4*_	Score (γ)
*Seq*_*7*_	223	87	0.5	7.66	*0.90*
*Seq*_*3*_	195	72	0.5	11.47	*0.80*
*Seq*_*19*_	238	84	0.45	11.5	*0.70*
*Seq*_*12*_	136	54	0.33	11.47	*0.60*
*Seq*_*9*_	145	51	0.2	22.89	*0.50*
*Seq*_*2*_	160	50	0.57	22.89	*0.40*
*Seq*_*16*_	247	81	0.4	22.91	*0.30*

### Utilities Calculation

**FIGURE 9:**
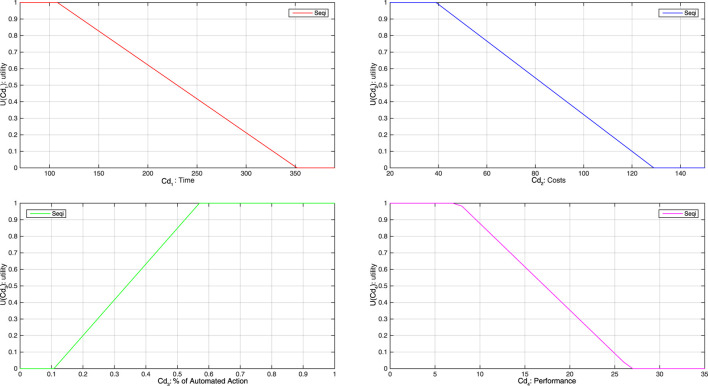
Utility functions.

The definition of utility functions will enable to map the criteria on a commensurable scale. Higher levels of utility are associated with the preferred values. Figure 9 shows the proposed functions.

The utility functions for each criterion are determined according to the following rule and considering the nominal state of the system. The minimum and maximum values of criteria are calculated considering all the operating sequences. The interval [0.2 1.0] is used for the utility of “nominal state” sequences. The use of these limit values is required since:

• no criteria can have a better value (the system is designed to be the best solution in its nominal state); hence the maximum value of the utility, i.e., 1, is given to these values,
• criteria may have worth values since when degrading, equipment may work in nonoptimal state but still good for the system operation. Hence, some nonzero utility values must be affected by these. That is why we use 0.2 utility value for the worth criteria values in order to calculate decision criteria with equipment health variations.

When operating in degraded scenario, criteria will take utility value of 0 showing not acceptable values.

### Aggregation

The parameters of the aggregation operator have to be identified. A subset of sequences $\left\{S\text{e}q_{7},\;S\text{e}q_{3},S\text{e}q_{19},S\text{e}q_{12},S\text{e}q_{9},S\text{e}q_{2},S\text{e}q_{16}\right\}$ is used for that purpose. The sequences of the subset are shown to an expert who has the following preferences:

$$S\text{e}q_{7}≻S\text{e}q_{3}≻S\text{e}q_{19}≻S\text{e}q_{12}≻S\text{e}q_{9}≻S\text{e}q_{2}≻S\text{e}q_{16}.$$

With respect to his preferences, the expert gives each of them a global score. The set of alternatives and the expert’s score are presented in Table 5.

The coefficients of the weighted arithmetic mean (WA) are calculated from the learning data provided by the expert, i.e. presented in Table 5. We resolve the system of equations A*W_i_ = B, where A is the alternatives matrix $\left[u_{cd_{1}},u_{cd_{2}},u_{cd_{3}},u_{cd_{4}}\right]$ and B is score vector [score]. Table 6 gives the obtained W_i_ coefficients.

**TABLE 6:** Coefficients of the WA operator.

	*W*_*1*_	*W*_*2*_	*W*_*3*_	*W*_*4*_
WA	0.026	0.234	0.097	0.642

**FIGURE 10:**
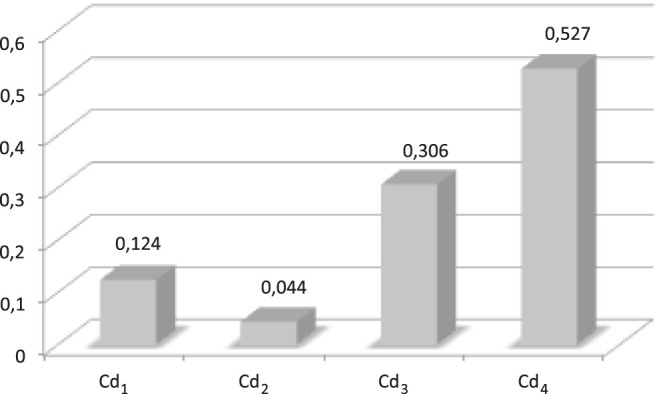
Shapley values for the CI operator.

**FIGURE 11:**
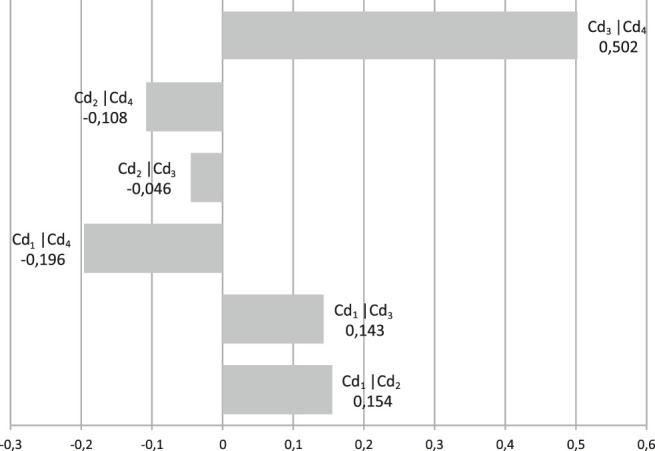
Interaction indexes for the CI operator.

Using the Kappalab extension of GNU-R software (Grabisch et al., 2007), the capacity is identified from the same learning data, i.e. Table 5. Figure 10 shows the Shapley values. Shapley's values give the importance of a criterion in relation to its contributions to overall capacity. For example, performance (cd_4_) has the highest weight in relation to other criteria such as with WA. The second most important criterion, for the WA, is cd_2_: cost. Figure 11 presents the interaction indexes for the identified capacity of the CI. The interaction indices reflect the interaction between two criteria across the capacity. Thus, a positive value of the interaction between a pair of criteria (e.g., cd_1_, cd_3_) corresponds to the configuration where when the utilities of cd_1_ and cd_3_ have a significant value, the CI result is greater than the result of the sum of the individual contributions. We then speak of synergy between the criteria. For a negative value of the interaction (e.g., cd_2_, cd_3_), an opposite interpretation is done. We then speak of redundancy between the criteria. We notice a strong positive interaction between (cd_3_, cd_4_), and a weaker interaction between (cd_1_, cd_2_) and (cd_1_, cd_2_). For the negative interactions, there are some between (cd_1_, cd_4_), (cd_2_, cd_3_), and (cd_2_, cd_4_).

### Ranking (on the learning set)

The obtained aggregation scores and sequences ranking for the weighted arithmetic mean (*WA*) and the Choquet integral (*CI*) operators are given in Table 7 for the learning subset of sequences. The rankings between expert preference, CI operator, and WA operator are:

$$S\text{e}q_{7}≻S\text{e}q_{3}≻S\text{e}q_{19}≻S\text{e}q_{12}≻S\text{e}q_{9}≻S\text{e}q_{2}≻S\text{e}q_{16}$$

$$CI\left(S\text{e}q_{7}\right)>CI\left(S\text{e}q_{3}\right)>CI\left(S\text{e}q_{19}\right)>CI\left(S\text{e}q_{12}\right)>CI\left(S\text{e}q_{9}\right)>CI\left(S\text{e}q_{2}\right)>CI\left(S\text{e}q_{16}\right)$$

$$WA\left(S\text{e}q_{7}\right)>WA\left(S\text{e}q_{12}\right)>WA\left(S\text{e}q_{3}\right)>WA\left(S\text{e}q_{19}\right)>WA\left(S\text{e}q_{2}\right)>WA\left(S\text{e}q_{9}\right)>WA\left(S\text{e}q_{16}\right).$$

Despite the small number of sequences, only CI operators have the ability to handle them properly. Thanks to the scores computed by CI operator, ranking results are similar to the expert preferences while the WA operator is not able to catch the expert preferences (for instance, Seq_12_ and Seq_2_ are not properly ranked). This result might not be surprising since WA has intrinsic limitation (Grabish and Labreuche, 2010). Such difference reflects in the mean square error which equals 5.57.10^−3^ for the CI and 55.82.10^−3^ for WA.

**TABLE 7:** Comparison of scores for the learning subset given by the expert, CI, and WA.

	*Expert*		*CI*		*WA*	
	score	rank	score (CI)	rang	score (WA)	rank
*Seq*_*7*_	*0,90*	1	0.877	1	0.848	1
*Seq*_*3*_	*0,80*	2	0.784	2	0.762	3
*Seq*_*19*_	*0,70*	3	0.736	3	0.714	4
*Seq*_*12*_	*0,60*	4	0.622	4	0.779	2
*Seq*_*9*_	*0,50*	5	0.460	5	0.375	6
*Seq*_*2*_	*0,40*	6	0.436	6	0.454	5
*Seq*_*16*_	*0,30*	7	0.293	7	0.327	7

But since the aim of the approach is to use the health checkup that reflects the component real status, what happens when the health of the components degrades? The next section presents the analysis of the model through the comparison of several operating conditions regarding the health status of the equipment.

### Scenario and Discussion

After checking the ranking on the learning set, look at the results on the complete set of sequences. Indeed, once the CI and WA parameters are learned, we can generalize the ranking to the whole set of sequences, i.e., $\left\{Seq_{1},\ldots ,Seq_{21}\right\}$. We also need to analyze the behavior of the ranking when the components will degrade, which is the main contribution of our proposal. Indeed, we compare the results of aggregation operators based on two “degradation” scenarios. Firstly, we consider a sequence with a slight deviation of equipment health indicators for the route R_1_. Secondly, we consider the larger deviation for the same route R_1_. Similarly, the expert proposes the same preferences for the ranking.

#### Ranking the Complete Set of Sequences in Nominal Mode

Table 8 shows the scores and ranking of the whole set of sequences in nominal mode, i.e., without considering degradation of equipment. Both rankings exhibit different results. The CI operators show more gradualness of the scores over all sequences. On the contrary, for WA operator, there is a clear discrepancy between the scores of $S\text{e}q_{20}$ and $S\text{e}q_{1}$; this discrepancy is not found in the scores of the CI sequences.

**TABLE 8:** Scores and ranking for the scenario in nominal mode for the CI and the WA operators.

	*IC*		*WA*	
*Rank#*	*Score (CI)*	*Seq#*	*Score (WA)*	*Seq#*
1	0,8768	7	0,8478	7
2	0,7994	5	0,8300	14
3	0,7844	3	0,8093	5
4	0,7844	6	0,7793	12
5	0,7678	21	0,7618	3
6	0,7361	19	0,7618	6
7	0,6891	14	0,7612	21
8	0,6887	17	0,7356	10
9	0,6887	20	0,7356	13
10	0,6223	12	0,7143	19
11	0,5572	10	0,6731	17
12	0,5572	13	0,6731	20
13	0,5216	8	0,4796	1
14	0,5216	11	0,4692	4
15	0,4851	1	0,4536	2
16	0,4851	4	0,4202	8
17	0,4598	9	0,4202	11
18	0,4364	2	0,3747	9
19	0,3529	15	0,3700	15
20	0,3529	18	0,3700	18
21	0,2927	16	0,3270	16

We also note, for both CI and WA, that some sequences have identical scores (e.g., 3 and 6, 15, and 18). Since R_1_ and R_3_ are identical, it is obvious that sequences, in which one is replaced by the other, obtain equal scores.

#### Scenario #1: Low Deviation of Health Indicators

For this first degradation scenario, we simulated a slight variation in the opening and closing times of the *VM_1_* manual equipment of the route R_1_. This degradation impacts only cd_1_. The new scores obtained for all the sequences are shown in Table 9.

**TABLE 9:** Scores and ranking for the scenario #1 for the CI and the WA operators.

	*IC*		*WA*	
*Rank#*	*Score (CI)*	*Seq#*	*Score (WA)*	*Seq#*
1	0,8746	7	0,8472	7
2	0,7972	5	0,8295	14
3	0,7844	6	0,8088	5
4	0,7823	3	0,7787	12
5	0,7656	21	0,7618	6
6	0,7339	19	0,7612	3
7	0,6891	14	0,7607	21
8	0,6887	20	0,7356	13
9	0,6865	17	0,7350	10
10	0,6223	12	0,7138	19
11	0,5572	13	0,6731	20
12	0,5572	10	0,6725	17
13	0,5216	11	0,4790	1
14	0,5130	8	0,4692	4
15	0,4851	4	0,4536	2
16	0,4765	1	0,4202	11
17	0,4598	9	0,4196	8
18	0,4364	2	0,3747	9
19	0,3529	18	0,3700	18
20	0,3443	15	0,3694	15
21	0,2927	16	0,3270	16

We obtain globally the same behavior as for the nominal scenario. We can note some changes in the ranking which are due to the fact that route R_1_ and route R_3_ are no longer equivalent. Thus, the scores obtained for the sequences using route R_1_ have slightly decreased. This decrease is normal since R_1_ equipment is degraded. The score must decrease. For example, for sequences $S\text{e}q_{3}$ and $S\text{e}q_{6}$, the score for the CI in nominal state is the same, 0.7844. It remains the same for $S\text{e}q_{6}$, since it does not include R_1_ equipment, while $S\text{e}q_{3}$ score becomes 0.7823 since it does include R_1_ equipment. The same behavior is observed for the WA. Thus, with a slight degradation, the order of the sequences slightly changes.

#### Scenario #2: Larger Deviation of Health Indicators

The second degradation scenario is intended to illustrate considering of a more serious degradation of the route R_1_. Thus, in addition to the first scenario degradation, we consider a leak at the level of the pipes T_x_ of route R_1_. This scenario thus consists in simulating not only a drop in the performance of the manual valve but also the malfunctioning of the pipes. Hence this scenario impacts cd_1_ and cd_4_. The new scores obtained for all the sequences are shown in Table 10. One can observe more significant changes in the order of the sequences. For instance, $S\text{e}q_{3}$ deacreases from rank #3 for the nominal scenario, to #4 for scenario 1 and #5 for scenario 2. The same is for $S\text{e}q_{15}$ which respectively to ranks #19, #20, and #21. The effect of the degradation on the overall score produces this decreasing in ranking. On the contrary, $S\text{e}q_{7}$ in the three scenarios remains ranked #1. Indeed, the difference of score with the second-best sequence was big enough to compensate the decreasing due to degradation.

**TABLE 10:** Scores and ranking for the scenario #2 for the CI and the WA operators.

	*IC*		*WA*	
*Rank#*	*Score (CI)*	*Seq#*	*Score (WA)*	*Seq#*
1	0,8690	7	0,8382	7
2	0,7844	6	0,8207	14
3	0,7698	5	0,7886	5
4	0,7599	21	0,7618	6
5	0,7540	3	0,7582	12
6	0,7213	19	0,7516	21
7	0,6887	20	0,7410	3
8	0,6836	14	0,7356	13
9	0,6739	17	0,7148	10
10	0,6223	12	0,6936	19
11	0,5572	13	0,6731	20
12	0,5446	10	0,6524	17
13	0,5216	11	0,4692	4
14	0,4851	4	0,4536	2
15	0,4598	9	0,4202	11
16	0,4364	2	0,3936	1
17	0,4332	8	0,3747	9
18	0,3967	1	0,3700	18
19	0,3529	18	0,3342	8
20	0,2927	16	0,3270	16
21	0,2646	15	0,2840	15

Moreover, thanks to other sequence score changes, $S\text{e}q_{6}$, whose score did not change, is now ranked #2 while it was ranked #3 with $S\text{e}q_{3}$ in nominal scenario and was ranked #3 alone in scenario 1.

We also notice that, for the sequences with the lowest scores, i.e., the last eight sequences, the order is completely changed. The impact of a degradation is more important on the scores when they are low. The same behavior is also observed for the WA.

Finally, Table 11 presents the scores of the sequences whose scores changed due to scenario 1 and scenario 2 degradation. We compute some statistics as well on these scores. First the CI operator gives a wider range to the scores than WA and both maximum and minimum values are over and above those of WA for all scenarios. The mean of the IC is lower than the mean of WA while for the standard deviation, the reverse is true. From these statistics, we can conclude that the scores of the IC are more grouped than those of WA while the queue of the cluster, i.e., extreme values, is further for the IC than for WA.

**TABLE 11:** Scores and ranking for both operators, CI and WA, for the three scenarios, i.e., nominal, #1 and #2, for the sequence including route 1.

	*IC*			*WA*		
	*nominal*	*Scenario1*	*Scenario2*	*nominal*	*Scenario1*	*Scenario2*
$Seq_{1}$	0,4851	0,4765	0,3967	0,4796	0,4790	0,3936
$Seq_{3}$	0,7844	0,7823	0.754	0,7618	0,7612	0,7410
$Seq_{5}$	0,7994	0,7972	0,7698	0,8093	0,8088	0,7886
$Seq_{7}$	0,8768	0,8746	0,8690	0,8478	0,8472	0,8382
$Seq_{8}$	0,5216	0,5130	0,4332	0,4202	0,4196	0,3342
$Seq_{10}$	0,5572	0,5572	0,5446	0,7356	0,7350	0,7148
$Seq_{12}$	0,6223	0,6223	0,6223	0,7793	0,7787	0,7582
$Seq_{14}$	0,6891	0,6891	0,6836	0,8300	0,8295	0,8207
$Seq_{15}$	0,3529	0,3443	0,2646	0,3700	0,3694	0,2840
$Seq_{17}$	0,6887	0,6865	0,6739	0,6731	0,6725	0,6524
$Seq_{19}$	0,7361	0,7339	0,7213	0,7143	0,7138	0,6936
$Seq_{21}$	0,7678	0,7656	0,7599	0,7612	0,7607	0,7516
*Range*	0,5239	0,5303	0,6044	0,4778	0,4778	0,5542
*Max*	0,8768	0,8746	0,8690	0,8478	0,8472	0,8382
*Min*	0,3529	0,3443	0,2646	0,3700	0,3694	0,2840
*Mean*	0,6568	0,6535	0,6244	0,6819	0,6813	0,6476
*Standard Deviation*	0,1528	0,1550	0,1798	0,1647	0,1648	0,1953

## Conclusion

This paper dealt with the problem of ranking operating sequences of a complex system. Indeed, operating sequence selection is performed using sequence generating method that does not consider the real state of the equipment. Such approaches are performed during the design phase of the critical system and lead to a set of few sequences to use during the operational phase. When a sequence has to be applied, one is selected among these few sequences thanks to the operating crew. The main drawback of such approach is mainly twofold. First, some sequences are ignored since they are discarded at the design phase. Second, humans can hardly consider tens, perhaps few hundreds, of equipment in a sequence neither the gradual performance drift due to equipment degradation. Both may lead to not selecting the optimal sequence to be applied. To overcome such issue, we propose an approach leveraging multiattribute utility theory with equipment health checkup. The main advantage of such approach is that it enables at the operating stage considering all the success sequences as well as all the equipment health in an objective way. In order to illustrate the performance of adopted tools, a case study is presented on the CISPI experimental platform.

This study presents the aggregation results obtained by the Choquet integral operator that allow ranking the operating sequences according to the expert’s preferences. This ranking is provided for different operating modes (nominal, deviation, and degradation) corresponding to different equipment health indicators. Our proposed approach has several advantages and shows potentialities in a complex industrial context. It allows also the formalization of knowledge and provides concise and overall information to expert. Moreover, this approach takes into consideration the heterogeneity, number, importance, solicitation, and actual condition of equipment in the decision making.

Future works will focus firstly on a comparative study between the results of the two methods presented in this paper and the ordered weighted averaging operator OWA (Filev and Yager, 1998). Beyond this comparison, the hierarchical aspect of health checkup should be integrated. Indeed, for complex systems, the health concept must be present at different abstraction levels and must reflect the existing relationships between these levels. Thirdly, we will apply this approach to other case studies (e.g., chemical complex processes) by considering several criteria associated with the system context with a more representative utility function. In addition, the utility functions learning aspect should be analyzed especially in the case of uncertain information.

## Data Availability

The datasets generated for this study are available on request to the corresponding author.

## Footnotes

a
http://safetech.cran.univ-lorraine.fr/

## Abbreviations

CI, Choquet integral; CISPI, Safe and interactive operating of industrial processes; MAUT, Multiattribute utility theory; WA/OWA, Weighted arithmetic mean/Ordered weighted averaging operators

## Nomenclature

γ: Score
≽: and ≥ Preference operator and Comparison operator
ψ: and ϕ Aggregation operator and Ranking operator
$ℰ\left(seq_{i}\right)$: Set of equipment considered in $seq_{i}$
$P\left(seq_{i}\right)$: Operating sequence properties
$v\left(eq_{i}\right)$: Set of $eq_{i}$ health indicators, health checkup of $eq_{i}$
A_i_: Actions
*CAM*_*j*_*/CAA*_*j*_: Manual/Automatic actions costs
*CdM*: Materials costs
cd_i_: Decision criterion
CU/TU,: Costs/Times unit
Cx/Tx,: Fluid tanks/pipes
D_n_/D_f_: Nominal/leak flow
$eq_{i}$/eq_j_^M^: Equipment/Manual equipment
FIi/PIi,: Flow/pressure sensors
$seq_{i}$: Operating sequence
T_o_/T_f_: Opening/Closing time
T_p_: Execution time
$u_{k}\left(\;\right)$: Utility function
V_MO_/V_Lg_: Tanks/Lineage volumes
VMi/VVi,: Manual valves
VRi/VEi,: Control/Solenoid valves
W_i_: Weights
┴ and ┬: valve opening and closing actions
